# Genome-Wide Analysis of *C-Repeat Binding Factor* Gene Family in *Capsicum baccatum* and Functional Exploration in Low-Temperature Response

**DOI:** 10.3390/plants13040549

**Published:** 2024-02-17

**Authors:** Yanbo Yang, Qihang Cai, Li Luo, Zhenghai Sun, Liping Li

**Affiliations:** 1 College of Geography and Ecotourism, Southwest Forestry University, Kunming 650224, China; yang_yb@swfu.edu.cn; 2 College of Landscape and Horticulture, Southwest Forestry University, Kunming 650224, China; caiqihang@swfu.edu.cn (Q.C.); luoli6221@swfu.edu.cn (L.L.); 3 Yunnan International Joint R&D Center for Intergrated Utilization of Ornamental Grass, International Technological Cooperation Base of High Effective Economic Forestry Cultivating of Yunnan Province, South and Southeast Asia Joint R&D Center of Economic Forest Full Industry Chain of Yunnan Province, College of Landscape and Horticulture, Southwest Forestry University, Kunming 650224, China; 4 College of Wetland, Southwest Forestry University, Kunming 650224, China

**Keywords:** pepper, cold stress, expression pattern, *CBF*, functional analysis

## Abstract

*Capsicum baccatum* is a close relative of edible chili peppers (*Capsicum annuum*) with high economic value. The *CBF* gene family plays an important role in plant stress resistance physiology. We detected a total of five *CBF* genes in the *C. baccatum* genome-wide sequencing data. These genes were scattered irregularly across four chromosomes. The genes were categorized into three groupings according to their evolutionary relationships, with genes in the same category showing comparable principles for motif composition. The 2000 bp upstream of *CbCBF* contains many resistance-responsive elements, hormone-responsive elements, and transcription factor binding sites. These findings emphasize the crucial functions of these genes in responding to challenging conditions and physiological regulation. Analysis of tissue-specific expression revealed that *CbCBF3* exhibited the greatest level of expression among all tissues. Under conditions of low-temperature stress, all *CbCBF* genes exhibited different levels of responsiveness, with *CbCBF3* showing a considerable up-regulation after 0.25 h of cold stress, indicating a high sensitivity to low-temperature response. The importance of the *CbCBF3* gene in the cold response of *C. baccatum* was confirmed by the use of virus-induced gene silencing (VIGS) technology, as well as the prediction of its protein interaction network. To summarize, this study conducts a thorough bioinformatics investigation of the *CbCBF* gene family, showcases the practicality of employing VIGS technology in *C. baccatum*, and confirms the significance of the *CbCBF3* gene in response to low temperatures. These findings provide significant references for future research on the adaptation of *C. baccatum* to low temperatures.

## 1. Introduction

Plants are often subjected to a variety of abiotic stresses during growth, such as cold, heat, drought, heavy metals, and salt stress, which can limit the geographic range of plants and reduce their productivity [1]. Low-temperature stress has a huge impact on plants, destroying their plasma membrane structure, reducing their photosynthesis capacity, and over-accumulating reactive oxygen species (ROS), which can retard or even stagnate their growth and development [2,3,4]. During the long evolutionary process, plants have developed a series of complex and efficient regulatory mechanisms to cope with the threat of low-temperature. With the continuous development of molecular biology technology, the mechanisms by which plants cope with cold stress have been gradually clarified [5]. At the physiological and biochemical level, plants generate a series of osmotic adjustment substances such as soluble sugars, proline, and polyamine compounds to stabilize the cell membrane structure and scavenge reactive oxygen species [6,7]. In addition, a large number of protein kinases and transcription factors play a role in cold stress signaling pathways [8]. For instance, the overexpression of the *IbbHLH33* transcription factor from sweet potato in tobacco significantly improved the plant’s cold tolerance [9]. Similarly, overexpression of the *MbWRKY40* transcription factor from apple in *Arabidopsis* led to the accumulation of low-temperature-responsive genes, thereby enhancing its cold tolerance [10]. Transient expression of the *PpBZR1* transcription factor in peach can increase sucrose content in tissues, reducing low-temperature damage to tissues [11].

The C-repeat binding factor (*CBF*) is a member of the DREB subgroup within the APETALA2/ethylene-responsive factor (*AP2/ERF*) family, characterized by a conserved AP2 structure [12,13]. The *CBF* transcription factors are rapidly induced by low temperature and bind to the CRT/DRE (C-repeat/dehydration-responsive motif) cis-element in the cold responsive gene (*COR*) promoter, which in turn induces downstream *COR* gene expression. This process assumes a crucial role in the physiology of low-temperature response and cold resistance in plants [14,15,16]. Functioning predominantly in the early stages of cold stress in plants, *CBF* genes enables plants to respond rapidly to cold stress [17]. In *Arabidopsis thaliana*, the transcriptional activity of *CBF1*/*2*/*3* becomes activated within 15 min of cold treatment, with transcript level increasing rapidly, peaking at 3 h. Concurrently, the transcript level of *COR*, a downstream target gene of the *CBF* genes, begins to rise at 2 h of treatment [14,18]. Although *CBF* family genes share similar functions, their expression patterns differ [19]. Under cold stress, *Arabidopsis CBF1* and *CBF3* exhibit earlier expression than *CBF2*. Moreover, *CBF2* exerts a negative regulatory effect on the expression of *CBF1* and *CBF3* [20]. *CBF* genes have been identified in a diverse array of plants, encompassing both monocotyledonous and dicotyledonous species, and are closely linked to responses to low-temperature stress. For instance, overexpression of the maize *ZmDREB1A* gene enhances drought and cold tolerance in transgenic *Arabidopsis* [21]. Expression of the *CBF* gene *LeCBF1* of induces the expression of the downstream target gene *COR* in transgenic *Arabidopsis*. However, the overexpression of *AtCBF3* in tomato does not confer enhanced cold tolerance [22]. In recent years, a large number of reports have indicated that the *CBF* gene family is involved in a wide range of stress tolerance physiological processes in plants. For example, the *CBF* gene family in *Vitis vinifera* is widely involved in fruit development, shoot dormancy, drought response, copper stress response, and downy mildew response [23]; the *CBF* gene family in tea plant is involved in heat stress response, drought response, and salt stress response [24]; The *CBF* gene family in *Betula platyphylla* is involved in a variety of abiotic stress responses including low-temperature stress [25]. It is evident that the biological functions of *CBF* genes vary between different species.

Pepper stands as a valuable cash crop, finding applications in the edible, medicinal, and spice industries [26,27,28,29,30]. Within the *Capsicum* genus, comprising nearly 40 species [31], only five—*C. annuum*, *Capsicum frutescens*, *Capsicum chinense*, *C. baccatum*, and *Capsicum pubescens*—have been domesticated for cultivation [32,33]. Noteworthy among them is *C. baccatum*, an essential germplasm resource recognized for improving the quality of edible chili peppers, owing to its abundant nutritional value and high stress tolerance [31,34]. Widely acknowledged as one of the most significant chili peppers in South America, *C. baccatum* possesses a robust cultivation history in countries such as Argentina, Brazil, Chile, and others [34,35]. Beyond its culinary importance, *C. baccatum* serves medicinal purposes in its native regions, particularly for treating arthritis, dyspepsia, and respiratory diseases [36,37,38]. In addition, recent studies have highlighted its potential in reducing cardiometabolic risk in rats, suggesting a possible role in preventing metabolic syndrome [39]. Despite its immense potential, the breeding of *C. baccatum* significantly lags behind that of other edible peppers [40]. Numerous studies have shown that *C. baccatum* and *C. annuum* have very low cross-compatibility [41,42,43]. Therefore, the identification of valuable genes and targeted improvement through molecular biology methods are of great importance in the breeding of *C. baccatum*.

As the demand for high-quality vegetables continues to rise, people are increasingly concerned about their health benefits while also pursuing high yields [44,45,46,47]. *C. baccatum* is an economically important vegetable with rich nutritional and potential health benefits [48,49]. *C. baccatum* exhibits the growth characteristics of being temperature-loving and cold-intolerant, resulting in slow growth and fruit drop when exposed to cold damage [50,51]. This cold-induced impact significantly jeopardizes both the yield and quality of *C. baccatum* [52]. Cold injury has become a critical factor limiting the production and popularization of *C. baccatum*. Therefore, understanding the cold resistance mechanisms of *C. baccatum* and cultivating cold-tolerant varieties are crucial steps in enhancing its production value. In this study, the *CBF* gene family in *C. baccatum* was comprehensively analyzed at the genome-wide level with a view to revealing the regulatory mechanism of the *CbCBF* genes under low-temperature stress. This study lays the foundation for understanding the response mechanism of *C. baccatum* to low temperature and also provides candidate genes for the breeding of pepper resistance.

## 2. Results

### 2.1. Identification and Characterization Analysis of the CBF Genes in C. baccatum

Based on the HMM file of *CBF*, *C. baccatum* was searched genome-wide, and the protein sequences of *Arabidopsis* and *C. annuum* CBF were used for alignment in *C. baccatum* genome-wide. After manual screening and removal of redundant genes, a total of five *CbCBFs* were identified. Physicochemical analysis revealed diverse amino acid structure for CbCBF, the length ranging from 162 amino acids (CbCBF1) to 332 amino acids (CbCBF2). The predicted molecular weights spanned from 18.4 kDa (CbCBF1) to 36.3 kDa (CbCBF2). The theoretical pI of all CbCBF proteins was less than 7, indicating their potential negative charge in an alkaline pH environment. The predicted instability indexes of all CbCBF proteins except CbCBF4 were greater than 40, signifying their predominantly unstable nature. Moreover, the grand average of hydropathicity (GRAVY) values for all CbCBF proteins were less than 0, indicating their hydrophilic nature, with CbCBF1 being the most hydrophilic. Subcellular predictions suggest that localization of CbCBF proteins in the nucleus and cytoplasm (Table 1).

### 2.2. Evolutionary Analysis of CbCBF Proteins

**Figure 1:**
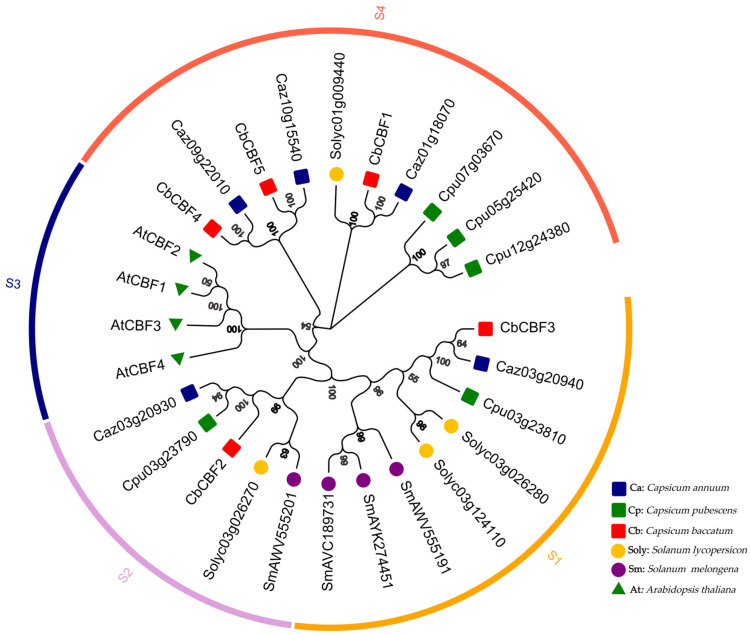
Evolutionary tree of the CBF proteins from six species. Different colors on the outside of the circle represent different subgroups (S1–S4). Different color and shape icons represent different species. The numbers in the figure represent bootstrap.

To investigate the evolutionary relationship of the CBF, an evolutionary tree of CBF proteins in *C. baccatum*, *Arabidopsis*, *C. annuum*, eggplant, tomato, and *C. pubescens* was constructed (Figure 1) and the evolutionary distances between these proteins were calculated (Table S1). The results showed that the evolutionary tree could be divided into four subgroups. *CbCBF* genes were distributed in three of these subgroups, with one *CbCBF* gene in the S1 subgroup, one *CbCBF* gene in the S2 subgroup, and three *CbCBF* genes in the S4 subgroup. All of the S3 subgroups were *Arabidopsis CBF* genes, and there were no *Arabidopsis* members distributed in the remaining subgroups. In addition, no eggplant *CBF* members were distributed in subgroup S4. *CbCBF* genes are evolutionary distant from *Arabidopsis CBF* genes. Among Solanaceae, *CbCBFs* are even more distant from eggplant *CBFs*. It can be concluded that the high stability of *CBF* genes during the evolution of Solanaceae played an indispensable and important role in life activities.

### 2.3. Conserved Motif Analysis of CbCBF Genes

**Figure 2:**
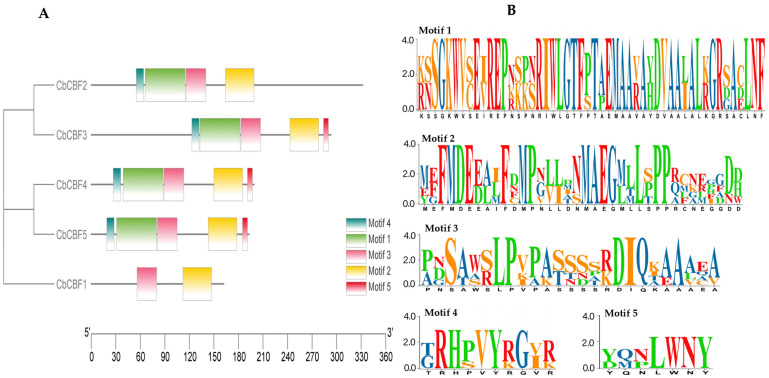
Conserved motif analysis of *CbCBF* genes. (**A**) Motif compositions. (**B**) Amino acid composition of each motif.

Motif analysis, facilitated by the MEME tool, was conducted, and the resulting motifs were visualized in conjunction with evolutionary relationships, as explicated in Figure 2A. Five conserved motifs were identified in total, with their sequences displayed in Figure 2B. Motifs 3 and 2 were found in all *CbCBFs*, while members of subgroup S1 and *CbCBFs* in subgroup S4 exhibited similar motif patterns, including motifs 5 exclusive to these two subgroups. This suggested a potential similarity in underlying functions for these two subgroups.

### 2.4. Cis-Acting Element Analysis of CbCBF Genes

**Figure 3:**
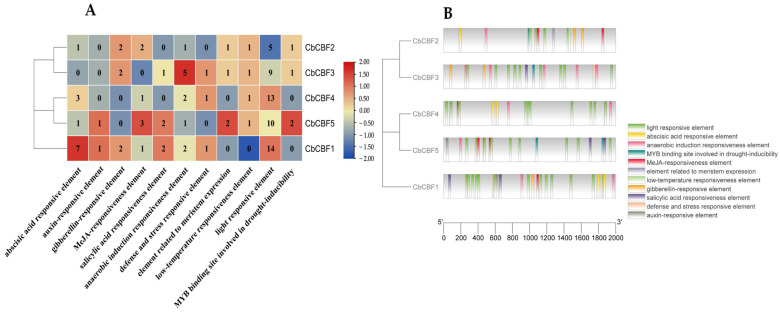
Analysis of cis-acting elements of *CbCBF* promoters. (**A**) The number of cis-acting elements of *CbCBF* promoters. (**B**) The position of cis-acting elements of *CbCBF* promoters.

To unravel the regulatory mechanisms of *CBF* genes in *C. baccatum*, the cis-acting elements within the 2000 bp promoter region upstream of *CbCBF* genes were analyzed. The findings revealed an abundance of stress-responsive, light-responsive, hormone-responsive, and transcription factor binding sites in the promoter region of *CbCBF* genes. The presence of light-responsive elements upstream of all *CbCBF* genes suggested potential regulation by light signals. All members except *CbCBF1* possessed low-temperature response elements, underscoring the critical role of the *CbCBF* gene family in response of *C. baccatum* to low-temperature. The hormone-response elements encompassed abscisic acid, auxin, gibberellin, MeJA, and salicylic acid response elements, indicating that *CbCBF* genes may be regulated by a complex network of hormones with implications for plant growth, development, and metabolism. Additionally, the drought-associated *MYB* transcription factor binding sites were identified in the promoter regions of *CbCBF2*, *CbCBF3*, and *CbCBF5*. It meant that these *CbCBF* genes may be regulated by *MYB* transcription factors in modulating the drought physiology of *C. baccatum*, as demonstrated in Figure 3.

### 2.5. Collinearity Analysis and Chromosomal Localization of CbCBF Genes

**Figure 4:**
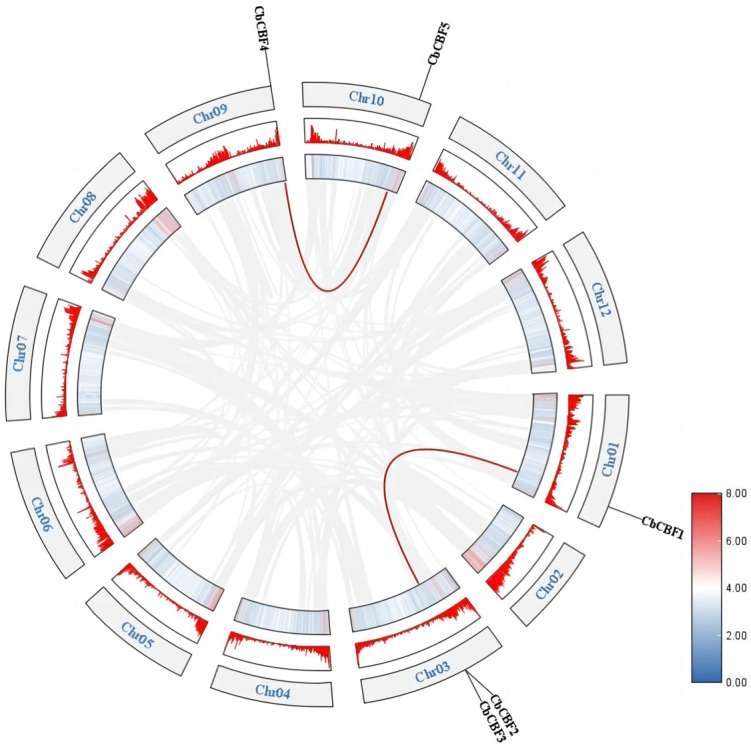
Collinearity analysis and chromosomal localization of *CbCBF* genes. The red line represents collinear genes. The color of the bars represents the gene density on the chromosome, with red indicating high density and blue indicating low density.

The chromosomal localization analysis revealed that *CbCBFs* are situated on four chromosomes: Chr01, Chr03, Chr09, and Chr10. The nomenclature of these members, namely *CbCBF1-5*, was based on their positional arrangement on the chromosomes. Notably, *CbCBF2* and *CbCBF3* were found to be tandemly arranged gene clusters. To delve into the origin and evolutionary relationships of *CbCBF* genes, the collinearity of *CbCBF* genes was analyzed, as illustrated in Figure 4. As it unveiled, in the *C. baccatum* genome, there were two pairs of *CbCBF* collinear genes: *CbCBF4*/*CbCBF5* and *CbCBF1*/*CbCBF2*. As a result, there were multiple duplication events within the *CbCBF* gene family, which contributed to the expansion of the *CbCBF* family. In addition, we calculated the nonsynonymous/synonymous substitution rates (Ka/Ks) in the covariate gene pairs, and the results showed that the Ka/Ks values were 0.127 in *CbCBF4*/*CbCBF5* and 0.281 in *CbCBF1*/*CbCBF2*, which is much smaller than 1. The Ka/Ks values were all much smaller than 1, so purifying selection played a major role in the evolutionary process.

### 2.6. Tissue-Specific Expression Patterns of CbCBF Genes

**Figure 5:**
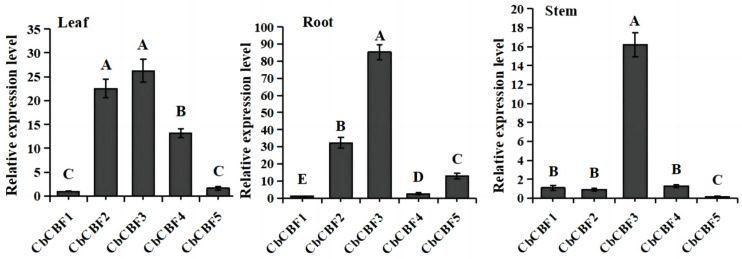
The expression patterns of *CbCBF* genes in different tissues. The *y*-axis represents the relative expression levels, the *x*-axis represents the different *CbCBF* genes. According to one-way ANOVA and Tukey’s tests (*p* < 0.01), different capital letters between samples indicated significant differences. The error bars represent the standard deviation.

To understand the regulatory ability of *CbCBF* in distinct plant parts, qRT-PCR analysis was employed to examine the expression levels in the in roots, stems, and leaves of *C. baccatum*. The findings, illustrated in Figure 5, revealed intricate expression patterns within the *CbCBF* family across diverse tissues. Specifically, *CbCBF2* and *CbCBF3* exhibited remarkably higher expressions in contrast to other *CbCBF* genes in leaves and roots, the expression level of *CbCBF3* gene in stems was significantly higher than that of other *CbCBF* genes. The *CbCBF1* and *CbCBF5* consistently demonstrated lower expressions compared to the other family members in various tissues. Notably, the relative expression of *CbCBF3* was the highest in all the tissues, indicating its pivotal role in the environmental adaptation of *C. baccatum*.

### 2.7. Expression Patterns of CbCBF Genes in Cold Stress

**Figure 6:**
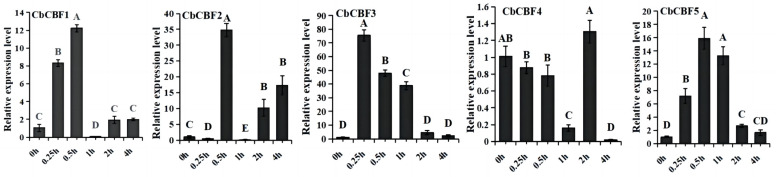
Expression patterns of *CbCBF* genes under different time points of 4 °C cold stress. The *y*-axis represents the relative expression levels, and the *x*-axis represents the time points of cold stress. According to one-way ANOVA and Tukey’s test (*p* < 0.01), different capital letters between samples indicated significant differences. The error bars represent the standard deviation.

To investigate the response of *CbCBF* genes to low temperature, we examined the expression levels of *C. baccatum* under low-temperature stress (4 °C) 0 h, 0.25 h, 0.5 h, 1 h, 2 h, and 4 h by qRT-PCR to understand the expression pattern of *CbCBF* genes under low temperature. The results showed that all *CbCBF* genes responded to low temperature to different degrees. Under low-temperature stress, *CbCBF1* and *CbCBF5* showed a significant response at 0.25 h, and the relative expression reached the highest at 0.5 h, after which the relative expression decreased significantly. *CbCBF2* increased significantly at 0.5 h of the low-temperature treatment. *CbCBF3* demonstrated a pronounced elevation in expression at 0.25 h, followed by a gradual decrease, ultimately returning to pre-low-temperature treatment levels at 2–4 h. In contrast, *CbCBF4* experienced a noticeable decrease in its expression at 0–1 h post-low-temperature treatment and elevated greatly at 2 h. It can be seen that different *CbCBF* genes respond to low temperature with different mechanisms. It is noteworthy that the expression level of *CbCBF3* gene increased significantly and reached the highest level at 0.25 h of low-temperature treatment, and then declined gradually after sustained cold stress, and its sensitivity to response to low-temperature was the highest within the *CbCBF* family, implying that it has an important role in cold stress and the early sensing of low temperature (Figure 6).

### 2.8. Virus-Induced Gene Silencing in C. baccatum

**Figure 7:**
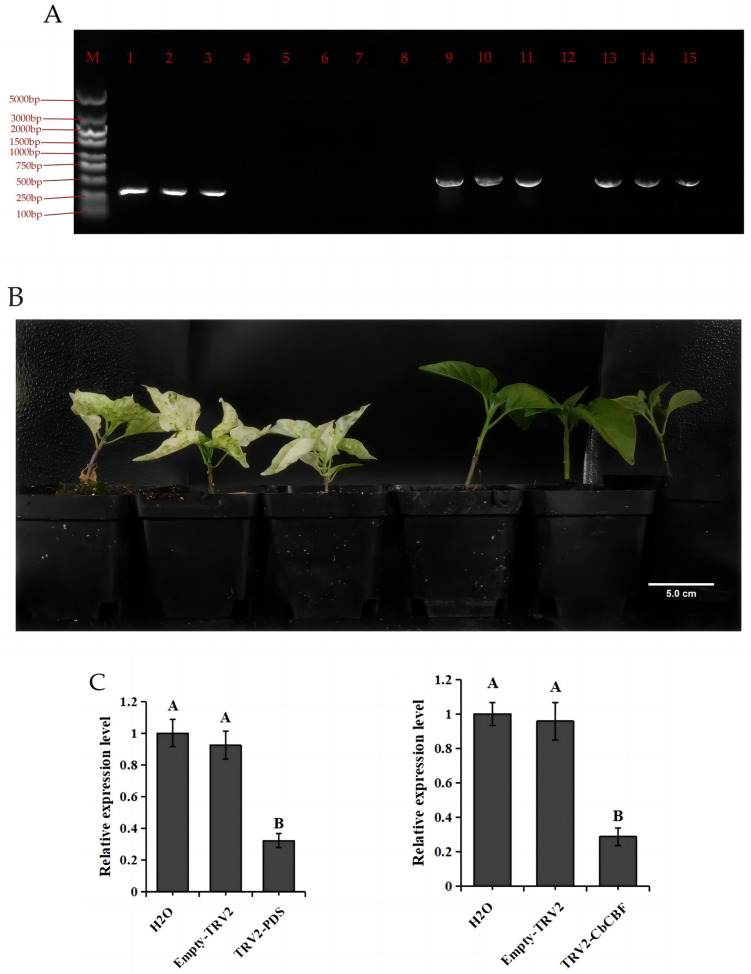
Virus-induced gene silencing in *C. baccatum*. (**A**) Gel electrophoresis of different silencing treatment groups. M: 5000 bp marker, 1–3: Empty TRV2, 5–7: H_2_O, 9–11: TRV2-*CbCBF3*, 13–15: TRV2-*PDS*. (**B**) Silencing of the *PDS* gene resulted in leaves with the distinct albino trait, indicating the success of the silencing system. The planting pots used in the picture are directly 10 cm. (**C**) qRT-PCR demonstrated that silencing significantly decreased relative gene expression levels. According to one-way ANOVA and Tukey’s test (*p* < 0.01), different capital letters between samples indicated significant differences. The error bars represent the standard deviation.

Combining the outcomes from the tissue-specific expression analysis and low-temperature stress expression modeling, the potential involvement of the *CbCBF3* gene was identified in the response of *C. baccatum* to low temperatures. To delve deeper into its role in the cold stress response of *C. baccatum*, the VIGS experiment was employed to silence the *CbCBF3* gene. To ensure experimental rigor, four distinct treatment groups were set up, namely H_2_O, Empty-TRV2, TRV2-*PDS*, and TRV2-*CbCBF3*. The results on the 18th day of silencing revealed that the leaves in the TRV2-*PDS* treatment group showed distinct albino traits (Figure 7B). PCR assay using TRV2 primers confirmed successful virus transmission in the Empty-TRV2, TRV2-*PDS*, and TRV2-*CbCBF3* treatment groups, with amplified bands indicating the presence of the virus. Notably, the amplified fragments of TRV2-*PDS* and TRV2-*CbCBF3* were approximately 300 bp longer than the amplified fragment of Empty TRV2 (Figure 7A), signifying the successful transmission of silenced fragments with the virus. Gene expressions in different groups were detected by qRT-PCR, with results displayed in Figure 7C, the VIGS significantly reduced the expression levels of *PDS* and *CbCBF3*.

### 2.9. Silencing the CbCBF3 Gene Reduces Cold Resistance in C. baccatum

**Figure 8:**
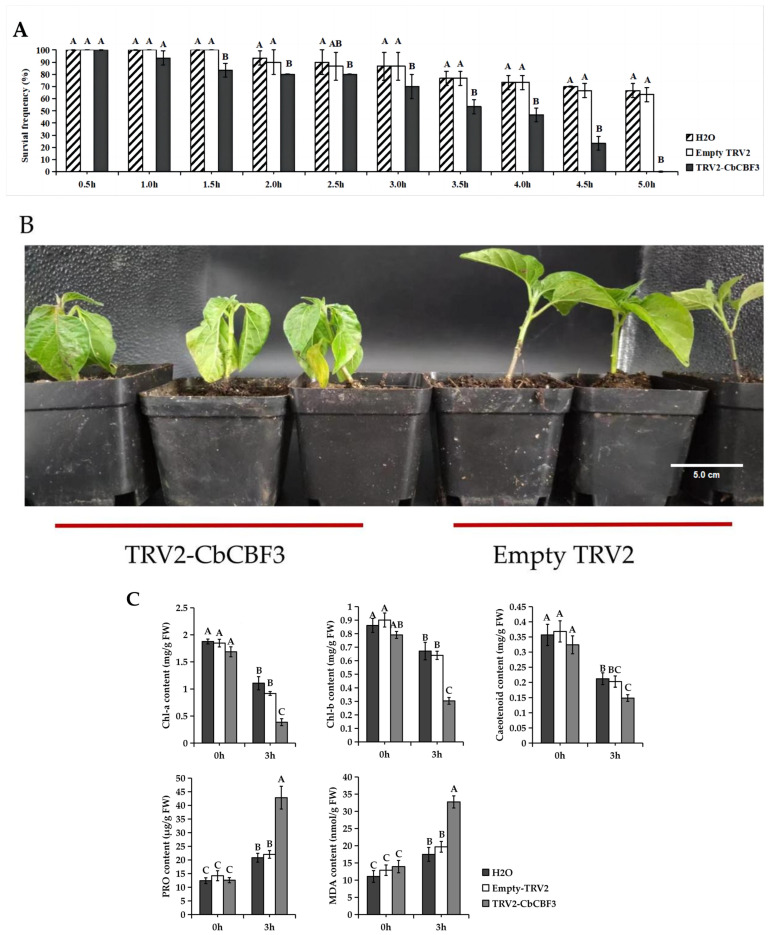
Effect of silencing of *CbCBF3* on *C. baccatum* under cold stress. (**A**) Plant survival was measured at different times of treatment at 4 °C. Each treatment group consisted of three replicate groups, with 10 plants per replicate group. (**B**) Phenotype of *CbCBF3*-silenced and control plants challenged with cold stress at 0.5 h post-treatment. The planting pots used in the picture are directly 10 cm. (**C**) Physiological indicators of *CbCBF3*-silenced and control plants challenged with cold stress at 3 h post-treatment. Chl-a: chlorophyll a; Chl-b: chlorophyll b; PRO: proline; MDA: malondialdehyde; FW: fresh weight. According to one-way ANOVA and Tukey’s test (*p* < 0.01), different capital letters between samples indicated significant differences. The error bars represent the standard deviation.

To examine the impacts of *CbCBF3* silencing on the low-temperature tolerance of *C. baccatum*, silenced plants were subjected to low-temperature treatment at 4 °C, with the plant survival rate monitored at 0.5 h intervals (Figure 8A). It was evident that survival rate in each treatment group experienced a decreasing trend with the increase of treatment duration under low-temperature stress. Noticeably, the survival rates of the H_2_O and the Empty TRV2 treatment groups did not exhibit sharp differences at each time point, suggesting that the transmission of tobacco rattle virus did not markedly influence the ability of *C. baccatum* to tolerate low temperature. In addition, plants silencing the *CbCBF3* gene began to die after 1.0 h of low-temperature treatment. The survival rate of *CbCBF3* silenced plants at 1.5 h was notably lower in comparison to that of the control group, and this trend persisted at all subsequent time points. All *CbCBF3* silenced plants died at 5.0 h of low-temperature treatment, while the survival rate of control plants remained above 60%. Additionally, *CbCBF3*-silenced plants exhibited more pronounced wilting than control plants starting from 0.5 h of low-temperature treatment (Figure 8B). Physiological indices were measured for control plants and *CbCBF3*-silenced plants under low-temperature treatment. The results showed that at 3 h of treatment at 4 °C, the leaves of *CbCBF3*-silenced plants accumulated more proline (PRO) and malondialdehyde (MDA), while the content of photosynthetic pigments was significantly reduced (Figure 8C).

### 2.10. Protein Interaction Network of CbCBF3 in C. baccatum

**Figure 9:**
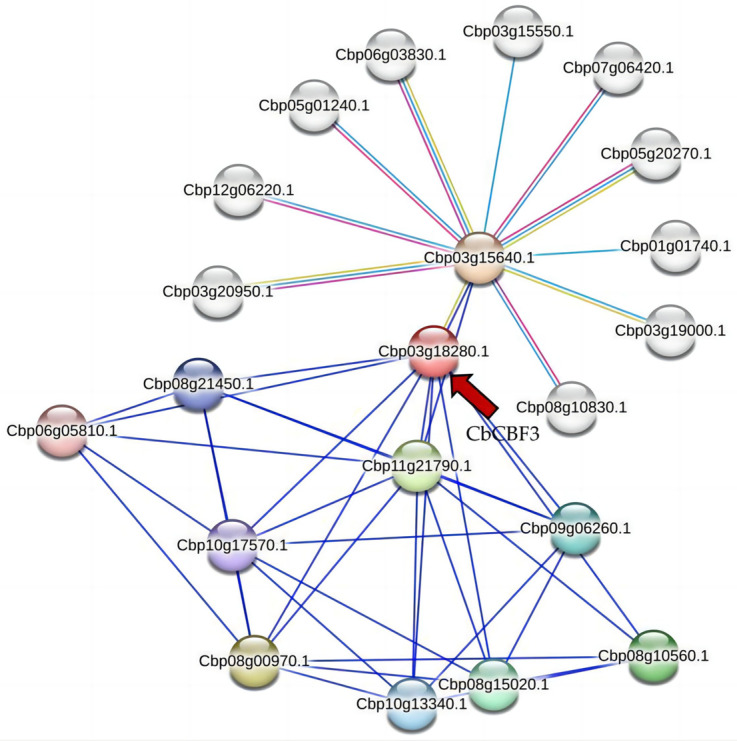
Interaction network analysis of CbCBF3 protein. Nodes represent different proteins, with colored nodes indicating proteins that may have a first shell of interactions with the CbCBF3 protein and uncolored nodes indicating proteins that may have a second shell of interactions with the CbCBF3 protein. Lines represent known or predicted protein–protein interactions. The red arrow is used to identify the CbCBF3 protein.

To further understand the function of CbCBF3 protein, its protein interaction network was predicted and analyzed, as demonstrated in Figure 9. CbCBF3 protein possessed potential interactions with abscisic acid receptor (Cbp03g18280.1), which can interact with several genes like Cbp12g06220.1, Cbp07g06420.1, Cbp05g01240.1, and Cbp08g10830. Further exploration through KEGG pathway prediction highlighted the involvement of these interacting proteins in the resistance-associated MAPK signaling pathway and the plant hormone signaling pathway. This observation suggests that CbCBF3 protein may play a role in regulating plant resistance physiology by interacting with proteins associated with abscisic acid regulation.

## 3. Discussion

Drought, salinity, low temperatures, and nutrient deficits are the primary environmental factors that hinder plant growth, development, and crop yields [53,54,55]. To cope with abiotic stresses, plants have developed stress response mechanisms at various levels—including morphological, physiological, biochemical, cellular, and molecular—over a long period of evolution. Among them, transcription factors play important roles in plant growth and development as well as in the response process to abiotic adversity stress, where a number of transcription factors are activated and subsequently a large number of defense-related genes are transcriptionally regulated, and these changes are essential for the establishment of defense mechanisms [56]. *CBF* transcription factors are important members of the *AP2/ERF* transcription factor family and are widely involved in a variety of plant responses to adversity [57]. In recent years, with the continuous progress of sequencing technology, the reference genomes of various plants have been increasing, and a large number of transcription factor gene families have been identified in different plants. *C. baccatum* is a plant with high potential value and low tolerance to low temperature [58]. However, the *CBF* transcription factors in *C. baccatum* is not well known, and the mechanism of their regulation in response to low temperature is poorly understood. Therefore, it is necessary to identify and systematically analyze the *CBF* gene family in *C. baccatum*.

In this study, bioinformatics analysis revealed the identification of five *CBF* genes in the genome of *C. baccatum*, exhibiting irregular distribution across four chromosomes. Previous reports have shown that the number of *CBF* genes varies among plants. For example, there are 8 *CBF* genes in potato [59], 3 *CBF* genes in cucumber [60], 16 *CBF* genes in walnut [61], and at least 15 *CBF* genes in cotton [62]. *C. baccatum* has fewer *CBF* genes compared to other plants, which may explain its weaker cold tolerance. The *CbCBF2* and *CbCBF3* genes formed tandemly arranged gene clusters, a phenomenon observed in the *CBF* family of various plants, such as *Arabidopsis* [63], cotton [62], and wheat [64]. Analysis of predicted physicochemical properties indicated that all CbCBF proteins are acidic, hydrophilic, and unstable proteins localized in the nucleus and cytoplasm. Evolutionary analyses showed that the *Arabidopsis CBF* family is evolutionarily more distantly related to *CbCBF*, and in Solanaceae, the *CbCBF* family is evolutionarily more distantly related to eggplant *CBF*. Previous studies have shown that tandem arrangement of *CBF* genes play important roles in family expansion and evolution [56], and their roles in the evolution of *C. baccatum* need to be further investigated. Collinearity analysis unveiled two pairs of segmental duplication genes within *CbCBF* members, with purifying selection being important in their evolution. In previous studies, it was noted that purifying selection eliminates deleterious mutations during the evolution of a species, thereby preserving ancestral functions in duplicated genes and therefore increasing the amount of ancestral gene products through dosage effects [57,58]. The *CbCBF* genes were all responsive to low temperature under low-temperature treatment, which may be influenced by a dose effect. This inference is consistent with previous studies in *Arabidopsis* [65]. Analysis of promoter cis-acting elements identified numerous light-responsive, hormone-responsive, and adversity-responsive elements upstream of the *CbCBF* genes. Previous studies have indicated the regulatory effect of light signaling on the *CBF* genes and the regular changes in expression of the *CBF* genes with light intensity throughout the day [66]. In addition, variations in light-quality affected *CBF* genes expression in plants, with the lower-red/far-red light ratio promoting its expression, enhancing the cold hardiness of plants at low temperatures. Therefore, it is inferred that the *CbCBF* genes may be regulated by light, thus influencing the cold hardiness of *C. baccatum*, and that its specific mechanisms need to be verified through further experiments.

The *CBF* gene functions in the early stages of cold stress in plants and enables plants to respond rapidly to cold stress [67]. Although the *CBF1/2/3* genes are functionally similar, the expression patterns are quite different [68]. In this study, we comprehensively analyzed the tissue-specific expression of *CbCBF* genes and the expression patterns under low-temperature stress, and the results showed that *CbCBF* genes had similar expression patterns in roots, stems, and leaves. The relative expression levels of *CbCBF1* and *CbCBF5* were low in all organs, and we believe that their expression must be induced by external factors, while *CbCBF3* had the highest expression level in all organs, and therefore may have an important regulatory function. *CbCBF* genes demonstrated complex expression patterns under low-temperature stress. Specifically, *CbCBF1* and *CbCBF5* were similar in this aspect, with a gradual increase under low-temperature treatment, peaking at 0.5 h, followed by a slow decline. In contrast, *CbCBF2* and *CbCBF3* rapidly increased and reached the highest level under low-temperature conditions. *CbCBF4* gene displayed a continuous decrease within 1 h upon this treatment but transiently increased at 2 h. As a result, *CbCBF* genes exhibited varying expression times under low-temperature stress, suggesting a feedback regulatory mechanism among *CbCBF* genes. Such a result is consistent with observations in *Arabidopsis* [63]. Notably, *CbCBF3* demonstrated the most significant increase in its expression at 0.25 h of low-temperature stress, aligning with the results of the tissue-specific expression analysis. It indicates that the *CbCBF3* gene greatly involves cold tolerance in *C. baccatum*.

VIGS serves as an approach to rapidly validate the gene function [69] and has been extensively applied in diverse plants, like tomato, tobacco, *Arabidopsis*, maize, loofah, cotton, and more [70,71,72,73]. The effectiveness of the VIGS system varies among plants due to different factors, such as virus–host interaction, plant growth status, and virus invasion [74]. Despite this, no previous studies have reported the application of VIGS technology in *C. baccatum*. In this research, VIGS analysis revealed a significant expression reduction in *PDS* gene in *C. baccatum* and obvious albino trait on the 18th day of silencing. These results demonstrated the feasibility of VIGS technology in *C. baccatum* for the first time. Subsequently, *CbCBF3* was silenced by VIGS technology to compute the survival rate of silenced plants at low-temperature conditions, verifying its important role in cold tolerance.

In addition, a predictive analysis was conducted on the CbCBF proteins interaction network, revealing interactions between the CbCBF protein and several abscisic-acid-regulated genes. Abscisic acid exerts a pivotal role in plant responses to environmental stress [75]. Previous studies have shown that abscisic acid and *CBF* are closely linked in signaling pathways and that abscisic acid can affect the CRT cis-acting element on the promoter of the *CBF* genes, thereby activating the expression of the *CBF* genes [38,76]. Under stress conditions, abscisic acid accumulation triggers the activation of the open stomata1 (*OST1*) gene, enhancing *CBF* genes expression [77]. This evidence suggests that the *CbCBF3* gene may engage in a complex and tight interaction with the abscisic acid pathway, regulating the stress tolerance physiology of *C. baccatum*.

## 4. Materials and Methods

### 4.1. Identification of CBF Genes in C. baccatum

The amino acid sequences of *Arabidopsis* CBF (AT4G25490, AT4G25470, AT4G25480, and AT5G51990) were sourced from the *Arabidopsis* genome database Tair (https://www.arabidopsis.org/) (accessed on 3 July 2023). The amino acid sequences of *C. annuum* CBF (Genome of Zhangshugang) (Caz01g18070, Caz09g22010, Caz03g20930, Caz10g15540, and Caz03g20940) were acquired from the pepper genome database (http://ted.bti.CORnell.edu/cgi-bin/pepper) (accessed on 3 July 2023). The genome-wide data of *C. baccatum* (Genome of PI_632928) were obtained from the pepper genome database (http://ted.bti.CORnell.edu/cgi-bin/pepper) (accessed on 3 July 2023). The identification of the *CbCBF* genes were involved two approaches. Firstly, the genome-wide of *C. baccatum* was searched by BlastP using the *C. annuum* and *Arabidopsis* CBF amino acid sequences. On the other hand, the HMM file of *CBF* (PF00847) was downloaded from the Pfam database (http://pfam.xfam.org/) (accessed on 3 July 2023) and the *CBF* genes were obtained from the *C. baccatum* genome using Tbtools v2.052 software [78].

### 4.2. Evolutionary Analysis of CBF Proteins

The *C. pubescens* CBF amino acid sequence (Genome of Grif_1614) (Cpu03g23790, Cpu03g23810, Cpu05g25420, Cpu12g24380) were downloaded using the pepper genome database (http://ted.bti.cornell.edu/cgi-bin/pepper) (accessed on 5 July 2023). The amino acid sequences (AWV55520.1, AYK27445.1, AVC18973.1, and AWV55519.1) of eggplant CBF were retrieved from the NCBI database (accessed on 5 July 2023). The amino acid sequences of tomato CBF (ITAG release 4.0) (Solyc03g026280, Solyc03g026270, Solyc03g124110, and Solyc01g009440) were obtained from the Sol Genomics Network (https://solgenomics.net/) (accessed on 5 July 2023). The amino acid sequences of CBF proteins in *C. baccatum*, *Arabidopsis*, *C. annuum*, tomato, eggplant, and *C. pubescens* were aligned by MEGA11 v11.0.10 software. The evolutionary tree was generated based on the neighbor-joining (NJ) approach, with the bootstrap method set to 1000. The evolutionary distance between each protein was calculated by MEGA11 11.0.10 software.

### 4.3. Genes Conserved Motifs Analysis

The amino acid sequences of CbCBF were analyzed utilizing the procedures mentioned in the MEME website (https://meme-suite.org/) (accessed on 5 July 2023) to obtain the motif annotation file. The genomic annotation file (.gff) was obtained from the *C. baccatum* genome database. Information on gene length and position was extracted from the genomic annotation file based on TBtools v2.052, with the analysis results visualized by the motif annotation file [78].

### 4.4. Cis-Acting Element Prediction

The genomic annotation file was analyzed using TBtools v2.052 for an extraction of 2000 bp sequence of the upstream promoter region of the *CbCBF* family. The promoter sequences were analyzed by referring to the procedures given by PlantCARE (https://bioinformatics.psb.ugent.be/webtools/plantcare/html/) (accessed on 9 July 2023), yielding a promoter annotation file. Hormone-responsive, adversity-regulated, environment-responsive, and transcription-factor-binding-site-related regulatory elements were retained. Finally, cis-acting elements were visualized using TBtools v2.052 software [78].

### 4.5. Collinearity Analysis

*C. baccatum* genome-wide collinearity relationships were analyzed using the MCScanX integration plug-in for TBtools v2.052 software [78]. The collinearity relationships were visualized using the Advanced Circos plug-in in TBtools v2.052 software to highlight the *CbCBF* gene collinear relationships. Then the *CbCBF* collinear gene pair nonsynonymous to synonymous substitution ratio was calculated using TBtools v2.052 software Simple Ka/Ks Calculator software [78].

### 4.6. Plant Materials and Treatments

*C. baccatum* (AjCB0071: A strain from *C. baccatum cv. “Bishop Crown”*) variety used in this experiment was planted in the experimental greenhouse of Southwest Forestry University (102.454308° E, 25.035229° N). The seeds were subjected to a 24 h soaking period in pure water and subsequently germinated on moist filter paper. After germination, the seeds were transplanted into seedling trays and then transferred to 10 cm diameter planting pots once they had grown mature leaves. The culture substrate used was a mixture of peat and vermiculite in a 3:1 ratio. Planting conditions were maintained at a temperature of 25 °C and a light cycle of 16 h per day. The plants were used for subsequent experiments upon reaching the stage of 4–6 leaves. The low-temperature stress treatment program was determined by referring to the experimental method of low-temperature treatment of peppers by previous researchers [79,80,81] and the expression pattern of *CBF* genes in other plants under a low-temperature environment [18,58]. Using an artificial climate chamber, the whole plant was subjected to low-temperature treatment. The temperature was maintained at 4 °C for varying lengths of time (0, 0.25, 0.5, 1, 2 and 4 h). Following the treatment, samples were carefully rinsed with ddH_2_O, soaked with liquid nitrogen, and kept at −80 °C for prolonged preservation.

### 4.7. Functional Analysis of CbCBF3 Based on VIGS

The VIGS experiment was modified based on the *C. annuum* VIGS system [42]. Four treatment groups were established—involving the injection of H_2_O, Empty-TRV2, TRV2-*PDS*, and TRV2-*CbCBF3*—to reduce the error of the VIGS experiment on the results. Agrobacterium tumefaciens (GV3101) carrying Empty-TRV2, TRV2-*PDS*, TRV2-*CbCBF3*, and TRV1 were cultured and resuspended to OD600 = 0.8. Equal volumes of TRV2 and TRV1 were mixed to create an infiltration solution. After being protected from light for 1 h, the seedling cotyledons were injected using a needleless syringe. The injected seedlings were then incubated in the dark at 24 °C for 24 h before being returned to the normal culture environment. Upon observing the albino trait in the TRV2-*PDS* treatment group, virus replication was assessed using TRV2 primers, and expression levels of target genes were determined by qRT-PCR. Subsequently, the silenced plants were subjected to cold stress treatment at 4 °C, and three parallel experiments were conducted for each treatment, consisting of 10 plants in each parallel group, and the survival rate was counted and recorded every 0.5 h.

### 4.8. Virus Vector Construction and qRT-PCR

A 300 bp silent fragment was selected using the VIGS site prediction website (https://vigs.solgenomics.net/) (accessed on 9 September 2023), and the target fragment was amplified using MegaFi™ Fidelity 2X PCR MasterMix (Applied Biological Materials Inc., Shanghai, China). Recombinant vectors were established utilizing the ClonExpress II One Step Cloning Kit (Vazyme Biotech Co, Ltd., Nanjing, China). RNA was extracted from tissues using FastPure Plant Total RNA Isolation Kit (Vazyme Biotech Co, Ltd., Nanjing, China), RNA integrity was examined using 1% agarose gel electrophoresis, and a nucleic acid proteometer (Thermo Fisher Scientific Inc., Waltham, MA, USA) was used to detect A260/A280 and RNA content (ng/μL). Reverse transcription of RNA to cDNA using All-In-One 5X RT MasterMix kit (Applied Biological Materials Inc., Shanghai, China). The actin gene (Cbp03g21510.1) was selected as the qRT-PCR internal reference genes using ArtiCanCEO SYBR qPCR Mix (Tsingke Biotechnology Co., Ltd., Beijing, China) for qRT-PCR. The qRT-PCR primers were designed by NCBI database primer-Blast function (Table 2). Relative gene expression levels were calculated using 2^−ΔΔCt^ [82].

### 4.9. Physiological Parameter Determination

The photosynthetic pigment content was determined using the spectrophotometric method [83,84]. To do this, 0.2 g of fresh leaf tissue was weighed and ground with 12 mL of 95% ethanol and a small amount of quartz sand until the tissue turned white. The mixture was then left for 5 min. The ground solution was filtered into a 25 mL brown volumetric flask using filter paper. The filter paper was rinsed with 95% ethanol, and the volume was adjusted to 25 mL with 95% ethanol. The solution was then shaken well. The absorbance of each sample was measured at 470 nm, 649 nm, and 665 nm, and the concentration of each photosynthetic pigment was calculated according to the following formula: Ca = 13.95A665 − 6.99A649, Cb = 24.96A649 − 7.32A665, Cx = (1000A470 − 2.05Ca − 114.8Cb)/245. Proline and malondialdehyde content were determined using kits provided by Comin Biotechnology Co. (Comin Biotechnology Co., Ltd., Suzhou, China).

### 4.10. Interaction Network Analysis of CbCBF3

Predictive analysis of the CbCBF3 protein interacting network was performed using the STRING website (https://cn.string-db.org) (accessed on 9 September 2023), using CbCBF3 as a lookup sequence for interacting protein prediction in the whole genome of *C. baccatum*. The potential regulatory mechanisms were analyzed based on the analysis results provided by the STRING website.

## 5. Conclusions

In this study, we identified five *CBF* gene family members in *C. baccatum*. The *CbCBFs* were subjected to evolutionary analysis, physical and chemical property prediction analysis, conserved motifs, gene structure analysis, and collinearity analysis. A large number of resistance-associated cis-acting elements were found in the promoter region of *CbCBF* genes. Expression pattern analysis showed that all *CbCBF* genes responded to low temperature. The cold-responsive function of *CbCBF3* was analyzed by VIGS technology, and its protein-interaction network was analyzed. The results indicate that *CbCBFs* play a significant role in *C. baccatum*’s response to adversity. This study provides a theoretical basis for breeding for improved cold tolerance in peppers.

## Figures and Tables

**Table 1:** Physicochemical analysis of CbCBF proteins.

Gene Name	Gene ID	Number of Amino Acid	Molecular Weight	Theoretical pI	Instability Index	Grand Average of Hydropathicity	Subcellular Localization
*CbCBF1*	Cbp01g28680	162	18,402.47	4.63	55.57	−0.701	Nucleus
*CbCBF2*	Cbp03g18270	332	36,252.64	5.96	62.57	−0.534	Cytoplasm, Nucleus
*CbCBF3*	Cbp03g18280	293	32,782.27	5.03	56.14	−0.33	Cytoplasm.
*CbCBF4*	Cbp09g26670	199	21,643.08	5.4	39.53	−0.476	Cytoplasm, Nucleus
*CbCBF5*	Cbp10g16750	193	20,203.37	5.68	50.39	−0.462	Nucleus

**Table 2:** The primer sequences used in this research.

Primer Name	Gene ID	Forward Primer Sequence (5′ –> 3′)	Reverse Primer Sequence (5′ –> 3′)
*CbCBF1*	Cbp01g28680	CAATTTAAGAGGAGGGCAGCTC	TGAAGAGCCGCGATTTGGAT
*CbCBF2*	Cbp03g18270	ATGGCGGAAGGGCTAATGTT	TACCGCTTCCGGGACAAAC
*CbCBF3*	Cbp03g18280	TCCCTACTGCTGAAATGGCG	ACTCTGATGGTCGGAAAGCC
*CbCBF4*	Cbp09g26670	ATGCGGATCTCAACTTCCCG	ACTGCTTCTTTACGTGCGGA
*CbCBF5*	Cbp10g16750	AGGCTGGAAATATGGCTGGG	TGCAGGAAGGTTGAGACGTG
*PDS*	Cbp05g00330	GGCTAAGGATTTCCGGCCTT	GACAAACCACCCAAACCTGC
*Actin*	Cbp03g21510	GGTCGGAATGGGACAGAAGG	GGTGCCTCCGTTAGGAGAAC
TRV	None	TGGGAGATGATACGCTGTT	CCTAAAACTTCAGACACG

## Data Availability

All data generated or analysed during this study are included in this published article.

## Supplementary Materials

The following supporting information can be downloaded at: https://www.mdpi.com/article/10.3390/plants13040549/s1, Table S1: Evolutionary distances for each protein in the CBF evolutionary tree.
